# Mapping Atrial Fibrillation Drivers

**DOI:** 10.31083/j.rcm2501026

**Published:** 2024-01-15

**Authors:** Yoshihide Takahashi

**Affiliations:** ^1^The Department of Cardiovascular Medicine, Shin-Yurigaoka General Hospital, 215-0026 Kawasaki, Japan

**Keywords:** atrial fibrillation, mapping, driver, focal, rotor

## Abstract

Understanding the initiating role of pulmonary veins in atrial fibrillation (AF) has led to the development of pulmonary vein isolation (PVI). The efficacy of PVI is high for paroxysmal AF, whereas it is limited for non-paroxysmal AF. This fact highlights the necessity of understanding the mechanism through which AF is maintained, to develop ablation strategies that would be required in addition to the PVI. Mapping AF in animal models and humans has led to the identification of focal or rotational drivers. New technologies have been developed to identify those AF drivers and are used as a guide for catheter ablation. This review article aims to provide a comprehensive overview of the current state of knowledge regarding AF drivers and the various mapping approaches used to identify them.

## 1. Introduction

The development of pulmonary vein isolation (PVI) has revolutionized the management of atrial fibrillation (AF). Despite the widespread use of PVI, the efficacy of this approach in a selected cohort of patients remains limited, such as the longstanding persistent AF or patients with advanced remodeled atria. To improve the efficacy of catheter ablation of AF, the identification of the mechanisms that maintain AF is crucial. Previous animal and clinical mapping studies have suggested that focal or re-entrant activations are driving AF, with those sites referred to as AF drivers. Mapping AF drivers has become a rapidly evolving field, and various mapping techniques and algorithms have been developed to localize and ablate those drivers. This review article aimed to provide a comprehensive overview of the current state of the knowledge regarding AF drivers and the various mapping approaches used to identify and target them.

## 2. Rotors

The debate regarding the AF mechanism began in the early 20th century. In 1920, Lewis proposed that AF was caused by single or multiple ectopic foci [1]. In 1964, Moe *et al*. [2] used a computation model to propose the “multiple wavelet hypothesis”. In 1994, Konings* et al*. [3] mapped AF in humans during cardiac surgery using a multi-electrode patch and reported re-entry and focal activation patterns during AF. The occurrence of re-entry and focal patterns appeared to be random. However, the limitation of that study was that mapping was performed during induced AF in non-AF patients, and the mapping area was limited to the free wall of the right atrium (RA).

In the 1990s, Jalife [4] developed an optical mapping technique that allows for high-resolution real-time mapping. Using this technique in an animal model, he discovered rapid and small re-entry during AF, known as rotors. In addition, he utilized fast Fourier transform (FFT) and phase mapping techniques to analyze the results of the optical mapping and demonstrated that there is a frequency gradient in the atria during AF, with the rotor existing in the highest frequency area. Based on those findings, he concluded that the rotor was driving AF and proposed the “mother rotor theory”. In the multiple wavelet theory, the atrial propagation patterns during AF are considered random. However, according to the mother rotor theory, the fibrillatory propagation is found to be organized within the disorganized electrical activity. Voltage-sensitive dyes are needed for optical mapping; however, voltage-sensitive dyes are toxic. Thus, this technique cannot be applied for mapping in humans.

## 3. Focal Activation

The development of a multi-spine electrode catheter (Pentaray, Biosense-Webster Inc., Diamond Bar, CA, USA) allowed for the analysis of 2-dimensional (2D) wavefronts propagation patterns during AF. The focal activation patterns during AF were identified by the Pentaray catheter [5]. In this study, mapping was performed before PVI in both paroxysmal and persistent AF patients. The mapping area that was recorded by the Pentaray catheter had a diameter of 35 mm, while six and four sites were mapped in the left atrium (LA) and RA, respectively, although not in the pulmonary veins (PVs), with the mapping time at each site recorded as 30 seconds. In this study, focal activity, lasting ≥3 consecutive atrial cycles, was observed in 9% of the mapping sites. Most of that activity was not sustained but appeared repeatedly at the same site during a 30-second recording period. This study demonstrated that focal activity exists in human AF. Focal activation during AF was deemed a driver of AF and potentially an optimal ablation target. However, catheter ablation targeting focal activation was not attempted because the analysis of the fibrillatory electrograms recorded by the Pentaray catheter was performed manually, which was time-consuming. Thus, software that could enable the identification of focal activations was desired for catheter ablation targeting AF drivers.

de Groot *et al*. [6] performed epicardial mapping of longstanding persistent AF and also found focal activation patterns. Of the focal activation, 90.5% were single events and only 0.8% consisted of repetitive focal activity that lasted >3 events. They investigated the unipolar electrogram morphology as well as the wavefront propagation patterns and identified an R wave during most focal activations, thereby suggesting that the mechanism underlying a focal activation pattern is the epicardial breakthrough of waves propagating in the deeper layers of the atrial wall. They also found that epicardial breakthroughs were more frequently observed in longstanding persistent AF than in acutely induced AF. Thus, they considered an epicardial breakthrough as a part of the re-entry circuit between the endo- and epicardium that maintains AF.

Although mapping was performed in a localized area in the abovementioned studies, Lee *et al*. [7] performed simultaneous biatrial epicardial mapping of human AF. They not only found epicardial breakthroughs that displayed an R wave in the unipolar electrogram but also ectopic foci that displayed a QS pattern in the unipolar electrogram. Ectopic foci were found at 2–4 sites in each patient and the focal activation was intermittent or sustained during a 32-second recording period. They also reported that no re-entry was found in that study.

## 4. Focal Impulse and Rotor Modulation (FIRM) Mapping

Narayan *et al*. [8] developed the focal impulse and rotor modulation (FIRM) mapping system, which enabled the real-time automatic analysis of atrial propagation patterns during AF. They used a 64-pole basket catheter to map the entire LA. The electrograms recorded by the basket catheter were analyzed using dedicated software that employed a phase map technique. FIRM mapping identified both focal and rotational activation, which remained stable over a long period of time. Catheter ablation was also performed and guided by FIRM mapping. The efficacy of FIRM-guided ablation was confirmed by acute responses to ablation, such as AF termination or the slowing of the AF cycle length. Furthermore, freedom from arrhythmia after the ablation was better than for the PVI-based conventional ablation strategy. However, FIRM had the following limitations. Some parts of the LA could not be mapped by the basket catheter. Additionally, given the size of the rotors observed in the animal models, the resolution of the mapping using a basket catheter seemed to be too poor. Importantly, the efficacy of the FIRM-guided ablation has not been validated by other groups [9, 10, 11].

## 5. Body Surface Mapping

Haissaguerre *et al*. [12] mapped AF using 252 body surface electrodes. After acquiring computed tomography images of the thorax, epicardial unipolar electrograms were reconstructed via a patient-specific biatrial geometry. This system uses the phase map technique as well as the FIRM system and shows focal or rotational activations. Catheter ablation was performed and guided by this system. The FIRM system showed that the focal or rotational activations were spatiotemporally stable, while the body surface mapping showed that the focal or rotational activations occurred intermittently, while the rotational activations meandered. Catheter ablation targeting the focal or rotational activations terminated AF in 80% of the persistent or longstanding-persistent AF patients. In contrast to the impressive acute AF termination rate, the long-term clinical outcomes were not as good as anticipated. At a 1-year follow-up, 59% of the patients were taking antiarrhythmic drugs, 64% were free from AF or atrial tachycardia, 16% had atrial tachycardia, and 20% had AF.

Phase mapping is used in both the body surface mapping system and FIRM mapping. This technique is helpful for identifying rotors. However, the phase mapping algorithm emphasizes rotational wavefronts. Therefore, phase mapping often shows false rotors particularly when cycle length varies or the signal-noise ratio is low [13], which are common in clinical settings. Although rotors identified by the body surface mapping system may be false, the diagnostic yield of this system has not been elucidated.

## 6. CARTOFINDER

**Fig. 1.**
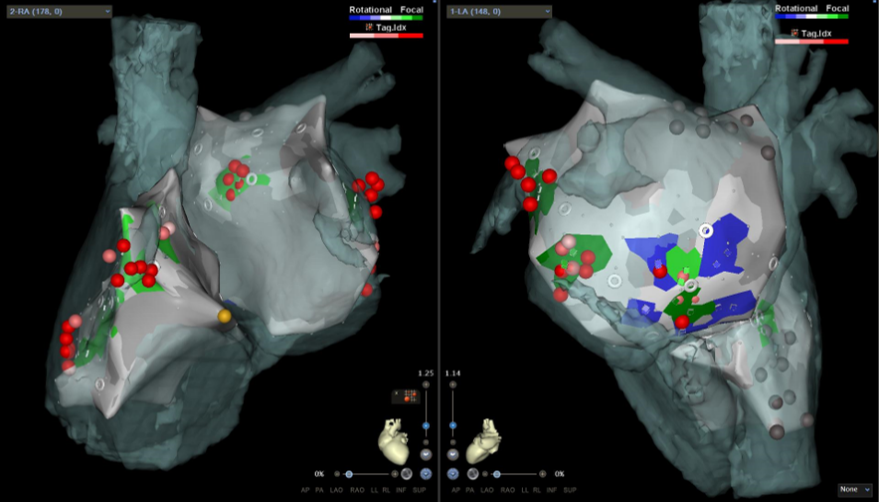
**CARTOFINDER map in a patient with a previous pulmonary vein isolation**. Although all of the pulmonary veins were isolated, this patient had recurring atrial fibrillation. Mapping using a Pentaray catheter was performed in both atria (left: anteroposterior view, right: posterior–anterior view). The green and blue areas represent focal and rotational activation, respectively. Ablation was performed by targeting the focal and rotational activation sites. LA, left atrium; RA, right atrium.

CARTOFINDER (Biosense-Webster, Diamond Bar, CA, USA) is a module dedicated to the CARTO system, which automatically identifies focal or rotational activation sites during AF, based on atrial electrograms recorded by a multielectrode catheter, such as the Lasso, Pentaray, or Octaray catheters (Biosense-Webster, Diamond Bar, CA, USA). The resolution of mapping using a Pentaray or Octaray catheter is higher than using a basket catheter, although the Pentaray or Octaray only covers a 30–40 mm area of the atrium. Therefore, to map the entire LA, mapping needs to be performed sequentially at multiple sites. Indeed, a 10- to 30-second electrogram recording is required at each site for analysis. The algorithm determines the local activation time from the unipolar electrograms. Focal or rotational activation sites are designated if the local activation time of each electrode in the catheter is consistent with a focal or rotational activation for at least two consecutive atrial cycles (Fig. 1). According to the CARTOFINDER map, a focal activation pattern is more often observed than a rotational activation pattern, and the most common site where focal activation is observed is the LA appendage [14, 15].

If focal or rotational activation sites are critical for AF recurrences after PVI, the presence of focal or rotational activation after PVI may predict the PVI clinical outcome. However, the post-PVI CARTOFINDER map was not associated with the clinical outcome of the PVI-alone ablation strategy [16]. The number of sites displaying focal or rotational activation was similar between the patients both with and without AF recurrences after PVI. Importantly, all patients who were free from atrial tachyarrhythmias after PVI alone had at least one focal or rotational activation outside of the PVs immediately after the PVI. This fact suggests that not all focal or rotational activation is critical for AF recurrence and that some are bystanders. Conversely, there are reports that ablation of focal activation sites is associated with AF termination [17], thereby suggesting that some focal activations play a role in the maintenance of AF. Chang *et al*. [18] reported that the clinical outcome of CARTOFINDER-guided ablation was better than that of PVI in a propensity score-matched non-paroxysmal AF patient cohort. However, no multicenter randomized controlled trials have assessed the efficacy of the CARTOFINDER-guided ablation.

## 7. Non-Contact Charge Density Mapping

**Fig. 2.**
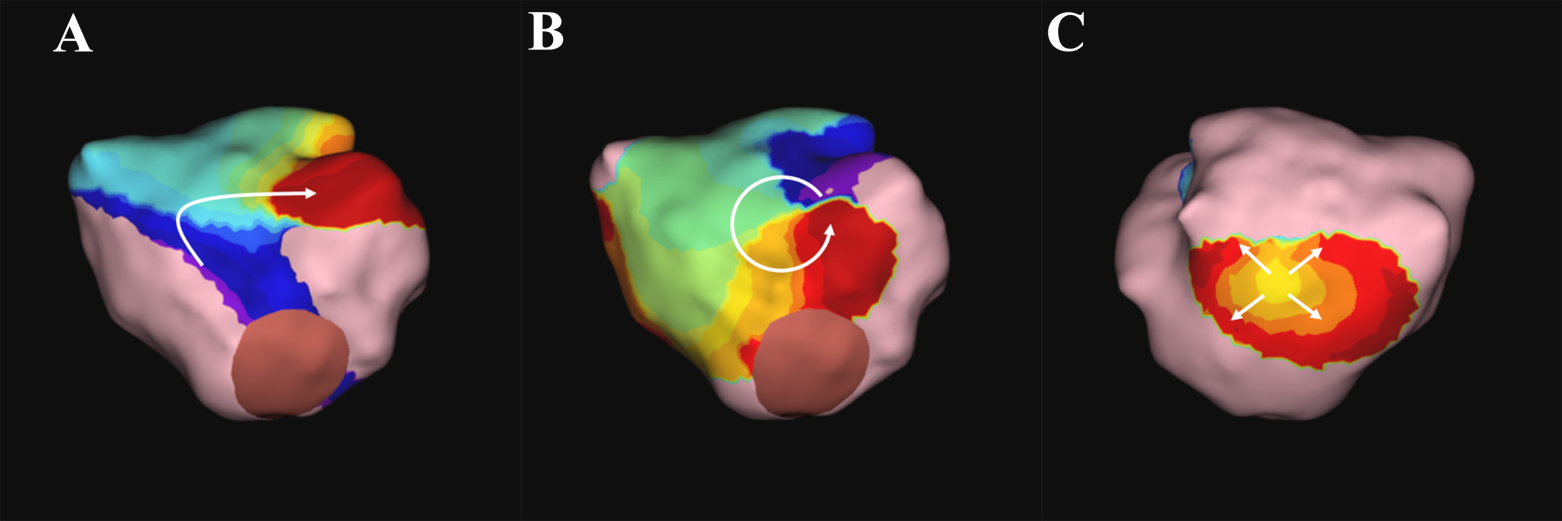
**AcQMap after pulmonary vein isolation**. The color denotes local activation timing. (A) Localized irregular activation, (B) rotational activation, and (C) focal activation. These figures were provided by Dr. Junichi Nitta.

The AcQMap system (Acutus Medical, Inc. Carlsbad, CA, USA) is a non-contact imaging and mapping system. The mapping catheter has 48 ultrasound transducers and 48 low-impedance high-fidelity electrodes. Ultrasound is used for the anatomic reconstruction, while high-fidelity electrodes are used to record the biopotential signals required to create the propagation maps. For an anatomic reconstruction, the electrodes do not need contact with the tissue. The geometry creation requires only several minutes. After the anatomic reconstruction, activation mapping is performed. The physicians do not need to move the catheter during the activation mapping. The activation map during AF using the AcQMap identifies the focal and rotational activation, similar to the other mapping systems. Additionally, localized irregular activation (LIA) is identified, which is where the activation displays a repetitive, multidirectional entry, exit, and pivoting conduction through and around a confined zone (Fig. 2). Compared to contact mapping, the mapping time is shorter with the non-contact mapping system. However, the locational accuracy and signal–noise ratio are relatively poor.

The UNCOVER AF trial (Utilizing Novel Dipole Density Capabilities to Objectively Visualize the Etiology of Rhythms in Atrial Fibrillation) was a single-arm, multicenter trial, which enrolled patients undergoing de novo ablation of persistent AF [19]. PVI was performed first and again after PVI, while any focal activation, rotational activation, and LIA were additionally ablated. At 1 year, the rate of freedom from AF, either on or off antiarrhythmic drugs, after a single procedure was 72.5%. More recently, the RECOVER AF study was reported that was also a single-arm, multicenter study [20]. In the RECOVER AF trial (Utilizing Novel Charge Density Capabilities to Objectively Visualize the Etiology of Recurrent Atrial Fibrillation Following a Failed AF Ablation), patients who underwent a repeat ablation procedure for recurrent AF were enrolled. The rate of freedom from AF, either on or off antiarrhythmic drugs, at 1 year after retreatment with the AcQMap was 76%.

## 8. Epi–Endo Asynchronous Activation

The development of new technologies increasingly allows us to understand fibrillatory propagation in the atria during ablation procedures. If we identify all AF drivers in real-time, ablating them all should theoretically result in the termination of AF. However, it remains challenging to terminate AF, even while guided by the new technologies. Furthermore, the arrhythmia-free rate after AF driver ablation in non-paroxysmal AF patients has not yet reached 90%. These facts may suggest that we do not fully understand the mechanism that maintains AF.

One of the limitations of the current mapping techniques is an inability to map the deep atrial layers or opposite surfaces. Simultaneous endo- and epicardial mapping has demonstrated that the endocardial propagation is often asynchronous with the epicardial activation during AF [21]. As mentioned above, an R wave is often displayed in unipolar electrograms at focal activation sites, thereby suggesting that wavefronts emerge from deep atrial layers that are asynchronously activated from the mapping surface. The incidence of breakthroughs becomes more prevalent as the AF duration increases in the animal models [22]. Thus, it seems that breakthroughs play an important role in the maintenance of AF. Furthermore, understanding the 3-dimensional (3D) atrial propagation may be key to improving the clinical outcome of non-paroxysmal AF.

## 9. Future Directions

Characteristics of the new mapping technologies are presented in the Table 1. The most critical issue regarding AF driver ablation is that the impact of AF driver ablation on the clinical outcome of catheter ablations has remained unclear up until now. Clinical trials are needed to address this issue. However, even if the efficacy of the AF driver ablation is proven, PVI will continue to be the cornerstone of the catheter ablation of AF, and AF driver ablation will be an option adjunctive to PVI in patients who are refractory to PVI alone. Therefore, patient selection for the AF driver ablation is an issue alongside identifying which patients should undergo AF driver ablation. Should it be performed in all non-paroxysmal AF patients? However, maybe not since PVI alone is only effective in at least half of the non-paroxysmal AF patients. No matter how well mapping systems become developed, there will be cases where AF occurs even after AF driver ablation. Thus, it is important to precisely predict the efficacy of the AF driver ablation in an individual patient, hopefully in a non-invasive manner.

**Table 1.** **Characteristics of the various mapping technologies**.

Mapping technologies	Mapping electrodes	Contact or non-contact mapping	Use of a phase map	Mapping area	Mapping findings
FIRM	64-pole basket catheter	Contact mapping	Yes	Entire atrial area	Stable focal or rotational drivers
Body surface mapping	252 body surface electrodes	Non-contact mapping	Yes	Entire bi-atria	Intermittent focal or rotational drivers
CARTOFINDER	Pentaray or Octaray catheter	Contact mapping	No	Localized area (sequential mapping required)	Intermittent focal or rotational drivers
Non-contact charge density mapping	48 low-impedance high-fidelity electrodes	Non-contact mapping	No	Entire atrial area	Intermittent focal or rotational drivers

FIRM, focal impulse and rotor modulation.

To improve the efficacy of the AF driver ablation, the accuracy of the mapping technologies needs to be improved. Additionally, the appropriate endpoint of the ablation also needs to be determined. In previous studies, it was reported that ablation of the focal or rotational activations, guided by new technologies, often terminated AF [12, 14, 17, 18, 19, 20]. However, the termination of AF is unlikely the optimal endpoint of the ablation because a previous study demonstrated that the termination of AF was not associated with a better clinical outcome [23]. Given that many patients are in AF at the end of the ablation procedure but are free from AF during the follow-up, the acute termination of AF is not necessary in those patients. Some patients have AF terminated during ablation, yet they have AF or atrial tachycardia recurrences during the follow-up. However, this is often observed after extensive complex fractionated atrial electrograms (CFAE) ablation and can be explained by the observation that the ablation of extensive atrial tissue creates a milieu, such as slow conduction or an isthmus for re-entry. According to the endpoint of the ablation, the efficacy of AF driver ablation may differ, even with the use of the same mapping technology.

## 10. Conclusions

In the last decade, various technologies have been developed for mapping AF. Although each new technology uses different techniques, such as contact mapping, non-contact mapping, and phase mapping, focal and rotational activation are identified during AF, regardless of the technology being used. This result is in line with the findings of the epicardial mapping of human AF. In recent studies, catheter ablation is performed that targets focal or rotational activation guided by new mapping technologies. However, the preliminary results of mapping-guided ablation of non-paroxysmal AF are not as high as the arrhythmia-free rate after PVI alone for paroxysmal AF. This may be due to not only the limitations of the mapping technologies but also to the fact that atrial propagation is asynchronous between the epi- and endocardial atrial layers during AF. While we are able to understand the propagation patterns on one side of the atrial surface, we need to further progress toward a complete understanding of the atrial propagation patterns during AF.
